# Phenolic Profiles, Antioxidant Capacity, and Enzyme Inhibitory Activities of *Helichrysum noeanum*: A Natural Source of Multifunctional Bioactive Compounds

**DOI:** 10.3390/molecules31132390

**Published:** 2026-07-07

**Authors:** Elif Aktürk Bozdemir

**Affiliations:** Department of Chemistry and Chemical Processing Technologies, Vocational School of Organized Industrial Zone, Tokat Gaziosmanpaşa University, Tokat 60200, Türkiye; elif.akturkbozdemir@gop.edu.tr

**Keywords:** *Helichrysum noeanum*, organ-specific bioactivity, phenolic profile, antioxidant capacity, enzyme inhibition, LC–ESI–MS/MS, Relative Antioxidant Capacity Index (RACI)

## Abstract

Natural bioactive compounds with antioxidant properties and associated biological activities are increasingly valued for food and nutraceutical applications. This study investigated the organ-specific phenolic composition, antioxidant capacity, and enzyme inhibitory activities of *Helichrysum noeanum* to evaluate its potential as a multifunctional source of such compounds. Methanolic extracts from flowers, leaves, stems, and roots were analyzed for total phenolics, flavonoids, and individual compounds using LC–ESI–MS/MS, alongside antioxidant and enzyme inhibitory activities. Leaves showed the highest phenolic content (87.23 mg GAEs/g extract) and strongest antioxidant capacity (RACI = 1.19), dominated by chlorogenic acid. Roots showed the highest concentrations of caffeic and ferulic acids, while stems contained benzoic acid derivatives and quercetin. Leaf extracts exhibited the most potent reducing and radical-scavenging activities, whereas roots and stems were superior in cholinesterase and α-amylase inhibition. Correlation analysis suggested that antioxidant responses were more closely associated with total phenolic content than with total flavonoid content, whereas enzyme inhibitory activities exhibited organ-dependent variation. Overall, *H. noeanum* represents an organ-specific source of bioactive compounds with potential relevance for nutraceutical and food-related applications.

## 1. Introduction

Natural products high in phenolic and flavonoid content are essential starting points for the development of pharmaceuticals, nutritional supplements, and cosmetic products due to their properties that influence redox balance, suppress enzymes, and reduce inflammatory processes [1,2]. Although phenolics are ubiquitous in plants, they are unevenly distributed among organs and tissues due to developmental/ecological factors; this variation often parallels organ-based antioxidant capacity and biological effects [1,3].

The genus *Helichrysum*, a member of the Asteraceae family, encompasses hundreds of taxa with a rich species diversity in Africa and the Mediterranean and has long been used in folk medicine [4,5]. Studies on this genus have shown an abundance of compounds such as phenolic acids and flavonoids, as well as pyrones, phloroglucinol derivatives, and terpenoids, which are associated with antioxidant and antimicrobial effects [4,6]. However, many species remain understudied, and modern, systematic profiling is needed to integrate traditional usage with mechanistic data [5,6].

*Helichrysum noeanum* Boiss., which has a limited distribution in Turkey, is a typical example of these shortcomings. The literature has largely focused on the essential oil and terpene profiles (γ-gurjunene, spathulenol, alloaromadendrene, β-caryophyllene, etc.) obtained from aerial parts; however, detailed examination of phenolic compounds in non-volatile extracts and comparisons of biological activity between organs have been rare [7,8]. However, the secondary metabolite repertoire can vary significantly among flowers, leaves, stems, and roots, which may lead to differences in measured antioxidant and enzyme inhibitory outputs and clinical relevance [9,10].

To reliably assess antioxidant potential, a multimethod panel measuring complementary chemistries is preferred. The Cupric Reducing Antioxidant Capacity (CUPRAC) assay and the Ferric Reducing Antioxidant Power (FRAP) assay, both based on electron transfer, measure reducing power; the 2,2-diphenyl-1-picrylhydrazyl (DPPH) radical-scavenging assay and the 2,2′-azinobis(3-ethylbenzothiazoline-6-sulfonic acid) (ABTS) radical cation scavenging assay evaluate radical-scavenging activity; the phosphomolybdenum method measures total antioxidant capacity; and iron(II) chelation assays assess the binding of pro-oxidant metals [11,12,13,14,15,16]. Because the chemical sensitivities of these assays vary, pooling the results, standardized by Relative Antioxidant Capacity Index (RACI), provides a unitless and comparable ranking across samples and is also consistent with individual assays [17].

Health-related enzymes also provide a strong rationale for screening herbal extracts. Simultaneous inhibition of acetylcholinesterase (AChE) and butyrylcholinesterase (BChE) provides symptomatic benefits in Alzheimer’s disease by increasing synaptic acetylcholine levels [18]. Inhibition of α-amylase and α-glucosidase contributes to diabetes management by reducing postprandial glycemia [19]. Tyrosinase inhibition has been used to control skin hyperpigmentation and enzymatic browning in foods [20]. These targets provide a meaningful translational framework for polyphenol-rich extracts.

In this context, the present study investigated methanolic extracts obtained from flowers, leaves, stems, and roots of *H. noeanum* at the organ level. Total phenolic/flavonoid amounts were determined; selected compounds were quantified by LC-ESI-MS/MS; antioxidant response was assessed by phosphomolybdenum, iron(II) chelation, DPPH, ABTS, CUPRAC, and FRAP assays; holistic ranking was performed by RACI; correlations among antioxidant assays were analyzed; and inhibition of AChE, BChE, α-amylase, α-glucosidase, and tyrosinase was screened. Despite the recognized organ-dependent distribution of plant secondary metabolites, no study has previously compared the phytochemical composition, antioxidant capacity, and enzyme inhibitory activities of different organs of *H. noeanum* within a unified experimental framework. Consequently, it remains unclear whether the bioactive potential of this species is uniformly distributed throughout the plant or concentrated in specific organs. Addressing this question is important both for understanding organ-specific phytochemical allocation and for identifying the most suitable plant materials for future bioactivity-oriented applications. Therefore, this study provides the first organ-level comparison of phenolic composition, antioxidant capacity, and enzyme inhibitory activities in *H. noeanum*, enabling evaluation of how different plant organs contribute to the overall bioactive profile of the species.

## 2. Results and Discussion

### 2.1. Phytochemical Composition

Before discussing the phytochemical composition and biological activities of the extracts, it is important to note the considerable variation in extraction yields among the investigated plant organs. Flowers provided the highest extraction yield (13.35%), followed by leaves (6.15%), roots (3.20%), and stems (1.97%). Extraction yield is an important parameter influencing process efficiency, raw material utilization, and the economic feasibility of large-scale production [21]. Therefore, organ-specific differences in extract productivity should be considered alongside phytochemical composition and biological activity when evaluating the practical potential of *H. noeanum* as a source of bioactive compounds.

**Figure 1:**
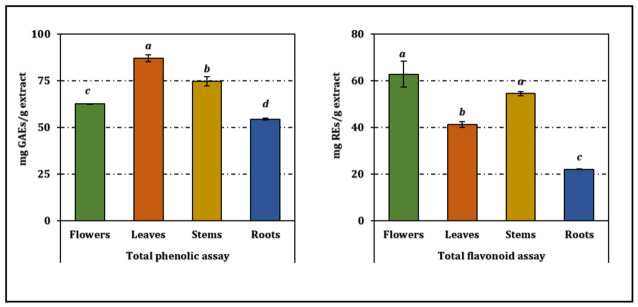
Total flavonoid and phenolic contents of *H. noeanum* extracts. REs and GAEs: Rutin and gallic acid equivalents, respectively. Values indicated by the same superscripts (a–d) within the same column are not significantly different according to Tukey’s HSD test at the 5% significance level.

Total phenolics and flavonoids varied markedly among organs (Figure 1). Leaves exhibited the highest phenolic burden (87.23 mg GAEs/g extract), followed by stems (74.86 mg GAEs/g extract), flowers (62.62 mg GAEs/g extract) and roots (54.49 mg GAEs/g extract). In contrast, flavonoids peaked in flowers (62.93 mg REs/g extract) and stems (54.58 mg REs/g extract), which were statistically indistinguishable, while leaves were lower (41.41 mg REs/g extract) and roots contained the least (22.15 mg REs/g extract).

Targeted profiling underscored chlorogenic acid as the leading phenolic across all extracts, maximized in leaves (8775 µg/g extract) and comparatively abundant in stems and flowers (Table 1). Distinct organ-specific signatures emerged: roots showed the highest concentration of caffeic acid (2755 µg/g extract) and showed the highest ferulic acid (65.1 µg/g extract), whereas stems concentrated multiple benzoic acid derivatives (protocatechuic, 3- and 4-hydroxybenzoic acids), vanillin, syringic acid, *p*-coumaric acid, and the flavonols quercetin and eriodictyol.

Flowers were the primary source of apigenin, apigenin-7-glucoside, luteolin-7-glucoside, and kaempferol, consistent with their elevated total flavonoid content. Kaempferol was not detected in stem and root extracts. For clarity, only compounds detected in at least one organ are presented in Table 1. Superscripts indicate statistically homogeneous groups according to Tukey’s HSD test (α = 0.05).

The present findings highlight distinct organ-specific differences in the phenolic and flavonoid profiles of *H. noeanum*, indicating organ-dependent variation in the quantified phenolic composition. The predominance of chlorogenic acid in all extracts, particularly in the foliar tissues, may reflect its important role in carbon flux through the phenylpropanoid pathway [22]. The higher abundance of this compound in leaves is consistent with its established function in photoprotection [23,24] and stress defense [25], commonly enhanced in photosynthetic organs. Similar trends have been reported for other *Helichrysum* species, where chlorogenic acid constitutes a major phenolic marker. For instance, *H. stoechas* methanolic extracts exhibited abundant chlorogenic and neochlorogenic acids [26,27], while *H. stoechas* and *H. sanguineum* also showed a strong presence of chlorogenic and caffeic derivatives [28,29].

The relatively high representation of caffeic and ferulic acids in the roots of *H. noeanum* may be linked to their structural and defense-related functions, as these compounds often reinforce root tissues and mediate allelopathic or antimicrobial interactions in soil environments [30,31,32]. The accumulation of benzoic acid derivatives and vanillin in the stems further indicates lignification and mechanical reinforcement, consistent with the reported occurrence of these compounds in *H. stoechas* [29].

Flowers, in contrast, exhibited a flavonoid-dominated profile typified by apigenin, apigenin-7-glucoside, luteolin-7-glucoside, and kaempferol. This compositional shift aligns with prior reports of abundant apigenin and its glycosides in *H. noeanum* methanolic extracts [33], as well as with other *Helichrysum* taxa, such as *H. stoechas* and *H. sanguineum*, where several flavonoids are consistently detected [26,28].

When compared to previous studies, the present organ-based approach expands the phytochemical understanding of *H. noeanum*. Previous phytochemical investigations of *H. noeanum* methanolic extracts reported chlorogenic acid, caffeic acid, ferulic acid, and apigenin derivatives as major constituents, although organ-specific differences were not evaluated in those studies [33]. The current data refine this picture by demonstrating that these compounds are unevenly distributed, suggesting organ-dependent variation in phenolic distribution rather than uniform metabolite partitioning. This observation aligns with reports from *H. stoechas*, in which different solvent fractions exhibited selective accumulation of hydroxycinnamic acids or flavonols depending on polarity [29].

The absence of certain compounds such as catechin, hesperidin, verbascoside, and rosmarinic acid in *H. noeanum* may indicate either species-level metabolic divergence or extraction-related specificity. While hesperidin and naringenin have previously been reported in *H. noeanum* extracts [33], their absence in the present dataset, despite the use of comparable solvent systems, may reflect organ-dependent distribution patterns or ecological variability among plant populations. Such chemical polymorphism is not uncommon within *Helichrysum*, as demonstrated by essential oil data showing considerable intraspecific variation in sesquiterpenes and oxygenated compounds [7].

Overall, the present work provides an organ-specific overview of phenolic distribution in *H. noeanum* and highlights marked differences among plant parts. The observed patterns mirror the general metabolic architecture of *Helichrysum* species and suggest organ-dependent variation in phenolic composition. These differences may be associated with the physiological and ecological roles of the organs and may influence the bioactivity profiles of specific plant parts. Future metabolomic and transcriptomic studies could further clarify the regulatory networks underlying these spatial patterns of secondary metabolism. From an applied perspective, identifying organ-specific phytochemical variation may facilitate the selective utilization of plant materials for antioxidant, nutraceutical, or enzyme-targeted applications, thereby improving resource efficiency and extraction strategies. Because the present study was based on targeted phenolic profiling of methanolic extracts, the observed organ-dependent differences should be interpreted within the scope of the analyzed compounds and extraction conditions rather than as a comprehensive representation of tissue metabolism.

### 2.2. Antioxidant Activity

The antioxidant capacities of *H. noeanum* extracts, expressed as IC_50_ or EC_50_ values, are summarized in Table 2. In general, the leaf extract consistently exhibited the strongest antioxidant potential across all assay systems, followed by the stem, flower, and root extracts.

In the total antioxidant capacity assay based on the phosphomolybdenum method, the leaf extract showed the lowest EC_50_ value (0.70 mg/mL), indicating superior reducing ability, while the root extract (0.84 mg/mL) and flower extract (0.85 mg/mL) were the least effective. A similar pattern was observed in CUPRAC and FRAP assays, where the leaf extract demonstrated the highest electron-donating and ferric-reducing power (0.55 mg/mL and 0.27 mg/mL, respectively), outperforming other plant parts.

In radical-scavenging assays, the leaf extract again stood out with the most potent activities, yielding IC_50_ values of 2.10 mg/mL for DPPH and 0.96 mg/mL for ABTS radicals, while the root extract displayed the weakest activity in both systems. In the metal chelation assay, the leaf extract also exhibited the strongest ferrous-ion binding ability (IC_50_ = 2.02 mg/mL), though all plant extracts were markedly less effective than EDTA.

**Figure 2:**
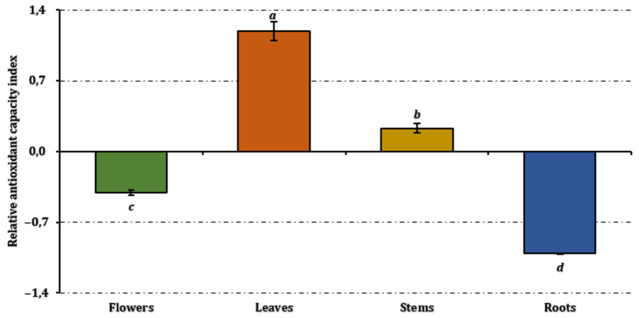
Relative antioxidant capacity index of *H. noeanum* extracts. Values indicated by the same superscripts (a–d) on the bar chart are not significantly different according to Tukey’s HSD test at the 5% significance level.

To provide an integrated comparison of antioxidant performance across assays, RACI values were calculated and are presented in Figure 2. The leaf extract showed the highest RACI score (1.19), followed by stem (0.23), flower (−0.41), and root (−1.01). Moreover, correlation analysis demonstrated strong concordance between RACI values and the individual antioxidant assays, indicating that extracts with higher phenolic contents generally exhibited stronger antioxidant performance.

The pronounced antioxidant potential of *H. noeanum* extracts, particularly in foliar tissues, highlights the organ-dependent variation in phytochemical composition and antioxidant performance within the *Helichrysum* genus. The strong reducing and radical-scavenging performances of the leaf extract are in line with its elevated total phenolic load, suggesting that the observed antioxidant patterns may be associated with the elevated total phenolic content of the leaf extract. Such organ-specific differentiation has not previously been reported for *H. noeanum*, although interspecific comparisons within the genus reveal similar trends linking phenolic abundance with antioxidant efficiency.

Earlier reports on *H. noeanum* have documented its antioxidant potential in whole-plant or aerial-part extracts without discriminating between organs. Strong DPPH radical-scavenging activity (IC_50_ ≈ 18 µg/mL) and high total antioxidant capacity (≈195 mg AAE/g extract) have previously been reported for *H. noeanum* extracts [33]. Similarly, substantial DPPH radical-scavenging activity (IC_50_ ≈ 45 µg/mL) and lipid peroxidation inhibition (≈57%) were reported for methanolic extracts of the species [34]. These earlier studies, although performed with different extraction protocols and assays, substantiate the current findings that *H. noeanum* exhibits a notable intrinsic redox capacity. The present results refine these insights by demonstrating that this capacity is not uniformly distributed but is concentrated in the photosynthetic tissues.

Comparative evaluation with other *Helichrysum* taxa further contextualizes the antioxidant profile of *H. noeanum*. For instance, *H. stoechas* extracts have shown strong radical-scavenging activity, with IC_50_ values around 10 µg/mL for DPPH and 5 µg/mL in lipid peroxidation systems [26,27]. Ethyl acetate extracts of the same species also exhibited potent hydroxyl radical quenching (IC_50_ = 0.18 mg/mL) [29]. Similarly, *H. sanguineum* exhibited solvent-dependent antioxidant variability, with the hexane and aqueous extracts showing stronger DPPH radical-scavenging activity than the methanolic and acetonic counterparts [28]. Despite methodological differences, these studies consistently emphasize that the genus *Helichrysum* represents a rich source of polyphenolic antioxidants, mainly flavonoids and phenolic acids.

The predominance of antioxidant activity in *H. noeanum* leaves may be associated with the higher abundance of phenolic constituents in photosynthetic tissues. Phenolic derivatives such as chlorogenic and caffeic acids, abundant in leaves of many *Helichrysum* species, have been reported to contribute to photoprotection and antioxidant defense under environmental stress conditions [23,24,25]. The lower activities recorded in roots may instead correspond to reduced phenolic diversity and a greater contribution of compounds associated with structural or ecological functions. This organ-dependent distribution is consistent with reports indicating that aerial tissues are generally exposed to higher levels of oxidative and photooxidative stress than belowground organs [23,24]. In addition to differences in phenolic abundance, variations in antioxidant activity among plant organs may also reflect qualitative differences in phenolic composition, interactions among constituents, and the presence of other non-phenolic metabolites that were not quantified in the present study. Similar observations have been reported in plant extracts, where antioxidant performance was influenced not only by total phenolic content but also by the specific composition of phenolic compounds and potential synergistic interactions among metabolites [10]. Therefore, antioxidant performance cannot be attributed solely to total phenolic levels, and multiple compositional factors may contribute to the observed organ-dependent responses.

Overall, the antioxidant potency of *H. noeanum* aligns well with the redox behavior reported for related *Helichrysum* species, yet the present study advances the understanding of its biochemical ecology by revealing tissue-specific differences. These organ-level variations not only mirror differential phenolic accumulation but may also guide selective utilization of specific plant parts for pharmacological or nutraceutical applications. Future metabolomic studies combined with activity-guided fractionation could further delineate the compounds or compound combinations associated with the observed effects, enabling the rational valorization of *H. noeanum* as a novel antioxidant source.

### 2.3. Enzyme Inhibitory Activity

The enzyme inhibitory activities of *H. noeanum* extracts are summarized in Table 3. Among the tested samples, the root extract exhibited the strongest inhibition against AChE (IC_50_ = 1.00 mg/mL), followed closely by the leaf and stem extracts (1.45 and 1.51 mg/mL, respectively). The flower extract showed the weakest AChE inhibition (5.58 mg/mL). For BChE, all extracts except the flower displayed comparable inhibitory effects, with IC_50_ values ranging from 1.16 to 1.25 mg/mL.

In the tyrosinase inhibition assay, all extracts demonstrated measurable inhibitory activity, with IC_50_ values clustered between 1.13 and 1.20 mg/mL, whereas kojic acid, the positive control, was markedly more potent (0.083 mg/mL). Regarding carbohydrate-hydrolyzing enzymes, the stem extract was the most effective α-amylase inhibitor (IC_50_ = 2.39 mg/mL), while the flower extract showed the lowest activity (3.69 mg/mL). In contrast, α-glucosidase inhibition was generally consistent across all extracts, ranging from 1.01 to 1.11 mg/mL, with values similar to that of acarbose (1.13 ± 0.01 mg/mL).

Overall, although none of the plant extracts matched the inhibitory efficiency of the reference compounds, the data highlight *H. noeanum*—particularly its root and stem extracts—as natural sources exhibiting multifunctional enzyme inhibitory activity.

The enzyme inhibitory findings obtained for *H. noeanum* represent the first report on this species, providing new insights into its potential as a multifunctional bioactive source. Although none of the extracts equaled the potency of standard inhibitors, the inhibition recorded for cholinesterases, tyrosinase, and carbohydrate-hydrolyzing enzymes indicates a diverse phytochemical arsenal capable of targeting multiple metabolic pathways.

The noteworthy inhibition of both AChE and BChE, particularly by root and leaf extracts, may be associated with phenolic or flavonoid derivatives previously reported to interact with cholinesterase enzymes. Comparable cholinesterase inhibition has been previously reported for *H. plicatum* and *H. chionophilum*, where ethanol and ethyl acetate extracts exhibited substantial AChE and BChE inhibition [35]. Moreover, methanolic extracts of *H. plicatum* subsp. *pseudoplicatum* displayed IC_50_ values in the low mg/mL range for AChE inhibition [36], consistent with the activity levels observed in *H. noeanum*. These observations collectively suggest that the genus Helichrysum contains secondary metabolites that may contribute to cholinesterase inhibitory activity. Such effects may be associated with compound classes previously reported in *Helichrysum* species, including flavonoids, phloroglucinols, and terpenoids [5,35,36]. Among the compounds detected in *H. noeanum*, flavonoids such as quercetin, kaempferol, apigenin, luteolin, and their glycosylated derivatives have previously been reported to exhibit acetylcholinesterase and butyrylcholinesterase inhibitory activities and may therefore contribute, at least in part, to the observed cholinesterase inhibition [37,38].

Tyrosinase inhibition by *H. noeanum* was comparable to that reported for related taxa such as *H. armenium* subsp. *armenium*, whose methanol and ethyl acetate extracts also showed moderate anti-tyrosinase potential [39]. Previous phytochemical investigations across *Helichrysum* species have identified quercetin, kaempferol, and their methoxylated analogs as compounds previously associated with melanogenesis-related enzyme inhibition [40]. The balanced tyrosinase inhibition exhibited by *H. noeanum* extracts may be associated with the presence of flavonoid scaffolds previously reported in *Helichrysum* species.

The α-amylase and α-glucosidase inhibitory activities further point to the antidiabetic relevance of this species. In *H. stoechas*, methanolic extracts showed α-glucosidase inhibition comparable to that of acarbose [26], whereas in *H. sanguineum*, aqueous and hexane fractions were particularly effective against α-amylase [28]. Similar trends were observed in *H. chionophilum* and *H. plicatum*, where several extracts exhibited dual inhibition of both enzymes [35]. The relatively balanced activity of *H. noeanum* across α-amylase and α-glucosidase may be associated with the combined contribution of phenolic constituents and other metabolites present in the extracts, including compounds that were not quantified in the present study.

Collectively, the enzyme inhibition data indicate that *H. noeanum* is a member of the *Helichrysum* genus exhibiting multitarget inhibitory activity against enzymes associated with neurodegenerative, dermatological, and metabolic disorders. The organ-dependent variation observed, particularly the higher AChE and α-amylase inhibition in root and stem tissues, underscores the influence of differential metabolite allocation across plant parts. Further phytochemical and mechanistic studies are warranted to isolate and characterize the constituents or combinations potentially associated with these effects and to explore their biological relevance in vivo.

It should also be recognized that the enzyme inhibitory activities observed in the present study may not be exclusively attributable to the phenolic compounds quantified by the targeted LC–ESI–MS/MS analysis. Plant extracts contain numerous additional metabolites, including both identified and unidentified constituents, that were not included in the analytical panel and may contribute individually or synergistically to the observed inhibitory effects. Therefore, the reported associations should be interpreted within the scope of the analyzed compounds while acknowledging the possible contribution of other bioactive metabolites.

### 2.4. Correlations Among Phenolic Compounds and Assays

It should be noted that the correlation analysis presented herein is exploratory in nature and does not imply causality. Because plant extracts are complex mixtures containing numerous identified and unidentified constituents, the observed biological activities are likely the result of combined, additive, or synergistic effects among multiple compounds. Therefore, the reported correlations should be interpreted as statistical associations rather than evidence that individual compounds are solely responsible for the observed activities.

In the correlation matrix (Table 4), the antioxidant assays showed strong internal concordance. Electron-transfer-based assays (FRAP and CUPRAC), radical-scavenging assays (DPPH and ABTS), and metal-chelating activity (FICA) generally exhibited similar response patterns. Likewise, RACI values closely reflected the results obtained from the individual antioxidant assays, supporting its suitability as an integrative measure of antioxidant capacity.

Total phenolic content showed strong positive associations with all antioxidant assays, whereas total flavonoid content exhibited comparatively weak relationships. These findings suggest that overall phenolic richness may contribute more substantially to the antioxidant performance of *H. noeanum* extracts than total flavonoid levels. Similar associations between total phenolic content and antioxidant activity have been reported for various medicinal and aromatic plants [10,41].

Enzyme inhibitory activities formed two distinct response patterns. Cholinesterase (AChE and BChE) and tyrosinase inhibition generally clustered together and showed positive associations with α-amylase inhibition, whereas α-glucosidase inhibition displayed a different trend. These observations may indicate that different groups of metabolites contribute to the various enzyme inhibitory responses observed among the investigated plant organs. In contrast to antioxidant activity, enzyme inhibitory responses showed weaker associations with total phenolic content, suggesting that factors other than overall phenolic abundance may contribute to the observed inhibitory effects.

## 3. Materials and Methods

### 3.1. Plant Material

*H. noeanum* was collected at full flowering stage on 1 August 2025, from gypsum-rich habitats near Kümbet Village, Zara District, Sivas Province, Türkiye (39°47′15″ N, 37°49′08″ E; 1895 m a.s.l.). The taxonomic identification was verified by Dr. Bedrettin Selvi, and a voucher specimen (GOPU 9611) was deposited in the Herbarium of the Faculty of Arts and Sciences, Tokat Gaziosmanpaşa University. Four anatomical parts of the plant—flowers, leaves, stems, and roots—were used in this study. Each organ was collected separately during harvesting. The samples were air-dried under shade in a well-ventilated environment protected from direct sunlight for approximately 3–4 weeks at ambient temperature (22–28 °C). Shade-drying was selected to minimize the thermal degradation and photooxidation of phenolic compounds that may occur under elevated drying temperatures or direct light exposure [21]. After drying, the samples were ground into a fine powder using a laboratory mill prior to extraction. All phytochemical and biological activity results are reported on an extract basis (mg or µg per g extract) rather than on a fresh- or dry-plant-material basis.

### 3.2. Methanol Extraction

Unless otherwise stated, all chemicals and reagents used in the experiments were of analytical or LC–MS grade purity. HPLC-grade methanol, the calibration standards gallic acid and rutin, and the phenolic reference compounds specified in the Supplementary Materials, as well as the reagents required for the total phenolic, total flavonoid, antioxidant, and enzyme inhibition assays, were supplied by Sigma-Aldrich (St. Louis, MO, USA) and Fluka (St. Louis, MO, USA). Reference standards that were not available from these two suppliers were procured from HWI Analytik (Ruelzheim, Germany). Formic acid of LC–MS grade was obtained from Merck (Darmstadt, Germany), whereas ultra-pure water was prepared in-house using a Milli-Q Plus purification system (Millipore, Bedford, MA, USA).

Dried samples were divided into four distinct organs (flowers, leaves, stems, and roots) and ground into a fine powder. Ultrasound-assisted extraction was performed in a sonication bath (Elma Schmidbauer GmbH, Singen, Germany) for 60 min using methanol as the extraction solvent, which was selected because of its well-established efficiency in recovering phenolic compounds from plant materials and its widespread use in phytochemical investigations [21]. A solid-to-solvent ratio of 1:20 (*w*/*v*) was employed following previously validated extraction protocols developed for comparable plant matrices [42]. The resulting extracts were concentrated under reduced pressure using a rotary evaporator (Heidolph Instruments, Schwabach, Germany) and stored at 4 °C until analysis. Prior to phytochemical and biological activity analyses, the dried extracts were re-dissolved in methanol at a concentration of 20 mg/mL and further diluted with methanol as required for individual assays. The extraction yields for methanolic extracts of *H. noeanum* flowers, leaves, stems, and roots were 13.35%, 6.15%, 1.97%, and 3.20% (*w*/*w*), respectively. Because no previous extraction optimization study has been conducted for *H. noeanum*, the selected extraction conditions should not be interpreted as optimal for this species. Rather, methanol and the applied extraction parameters were selected based on previously validated protocols widely used for phenolic-rich plant matrices and were applied uniformly to all plant organs to enable comparative evaluation under standardized conditions.

### 3.3. Determination of the Phenolic Compositions of the Extracts

Total phenolic and total flavonoid contents of the extracts were determined spectrophotometrically using a Shimadzu UV–Vis spectrophotometer (Shimadzu Corporation, Kyoto, Japan) according to previously reported protocols [43,44]. Calibration curves were generated using gallic acid and rutin as reference standards for total phenolic and total flavonoid determinations, respectively, and the results were calculated using the corresponding linear regression equations derived from the concentration–absorbance relationships. Calibration curves for total phenolic and total flavonoid determinations were constructed using gallic acid and rutin at concentrations of 10–100 µg/mL and 10–100 µg/mL, respectively.

Quantitative analysis of individual phenolic constituents was performed according to a previously validated LC–ESI–MS/MS method [45], adapted for the targeted determination of phenolic compounds in *H. noeanum* extracts. Quantitative analyses were performed on an Agilent Technologies 1260 Infinity liquid chromatography system coupled with a 6420 Triple Quadrupole mass spectrometer (Agilent Technologies, Santa Clara, CA, USA), using a Poroshell 120 EC-C18 column (100 mm × 4.6 mm, 2.7 μm particle size; Agilent Technologies, Santa Clara, CA, USA). Data acquisition and quantification were carried out using Agilent MassHunter Workstation software (Agilent Technologies, Santa Clara, CA, USA). Detailed chromatographic conditions, analytical parameters, calibration characteristics, and method validation data are provided in Tables S1 and S2 of the Supplementary Materials.

### 3.4. Biological Activity

Experimental protocols for antioxidant [46,47,48,49,50] and enzyme inhibitory activity assays [43,51] are described in detail in the Supplementary Materials. For antioxidant and enzyme inhibition assays, stock extract solutions (20 mg/mL) were serially diluted with methanol to generate concentration ranges appropriate for IC_50_ or EC_50_ determination. Depending on the assay, final extract concentrations ranged from 0.125 to 20 mg/mL. Detailed assay conditions, concentration intervals, and calculation procedures are provided in the Supplementary Materials.

### 3.5. Statistical Analysis

All analyses, including phytochemical profiling, spectrophotometric determinations, antioxidant assays, and enzyme inhibition assays, were performed in triplicate, and the results are expressed as mean ± standard deviation (SD). Statistical significance among means was assessed using one-way analysis of variance (ANOVA), followed by Tukey’s post hoc test, with a confidence level of *p* < 0.05. Analyses were performed using SPSS software (version 26.0).

Pearson’s correlation analysis was applied to explore relationships among measured parameters. Given the distinct reaction mechanisms involved in antioxidant assays, direct comparison of numerical values was not feasible. Prior to RACI calculation, antioxidant assay results expressed as IC_50_ or EC_50_ values were transformed using their reciprocal values (1/value), ensuring that higher transformed values consistently represented greater antioxidant capacity. The transformed values were subsequently standardized using z-score normalization and used for RACI computation [17].

## 4. Conclusions

This study demonstrated marked organ-specific differences in the phenolic composition, antioxidant capacity, and enzyme inhibitory activities of *H. noeanum*. Leaves contained the highest total phenolic content and exhibited the strongest antioxidant performance across the tested assays, whereas root and stem extracts showed comparatively higher cholinesterase and α-amylase inhibitory activities. The findings indicate that different plant organs possess distinct bioactivity profiles that may be relevant for the selective utilization of plant materials in antioxidant- and enzyme-targeted applications. Although the observed activities were lower than those of the reference standards, the results provide new information on the organ-level phytochemical and biological characteristics of *H. noeanum*.

Further studies are warranted to identify additional bioactive constituents and to confirm these findings using broader phytochemical and biological approaches.

A limitation of the present study is that residual moisture content was not determined after the shade-drying process. Although all plant organs were dried under identical conditions, potential differences in residual moisture among organs cannot be completely excluded and may have influenced extraction yields and organ-to-organ comparisons. In addition, extraction optimization was beyond the scope of the present study; therefore, the reported results should be interpreted as organ-specific comparisons obtained under a single standardized extraction protocol.

## Figures and Tables

**Table 1:** Concentration (µg/g extract) of selected phenolic compounds in *H. noeanum* extracts.

Compounds	Flowers	Leaves	Stems	Roots
Chlorogenic acid	7567 ± 30 *^b^*	8775 ± 70 *^a^*	7655 ± 34 *^b^*	4348 ± 32 *^c^*
Hyperoside	1385 ± 12 *^c^*	2159 ± 27 *^b^*	2960 ± 66 *^a^*	173 ± 4 *^d^*
Caffeic acid	376 ± 1 *^c^*	136 ± 1 *^d^*	415 ± 8 *^b^*	2755 ± 2 *^a^*
Luteolin 7-glucoside	615 ± 1 *^a^*	131 ± 6 *^c^*	206 ± 9 *^b^*	17.2 ± 0.8 *^d^*
Protocatechuic acid	33.8 ± 0.6 *^d^*	95.5 ± 2.1 *^b^*	328 ± 1 *^a^*	84.8 ± 2.0 *^c^*
3-Hydroxybenzoic acid	83.5 ± 0.1 *^b^*	54.0 ± 0.8 *^c^*	113 ± 1 *^a^*	22.3 ± 0.3 *^d^*
4-Hydroxybenzoic acid	83.6 ± 0.7 *^b^*	53.6 ± 1.4 *^c^*	107 ± 6 *^a^*	18.4 ± 0.6 *^d^*
Apigenin	1092 ± 10 *^a^*	40.7 ± 0.6 *^c^*	172 ± 4 *^b^*	22.0 ± 0.8 *^c^*
Apigenin 7-glucoside	751 ± 3 *^a^*	39.0 ± 1.3 *^d^*	118 ± 4 *^b^*	105 ± 1 *^c^*
Kaempferol	152 ± 1 *^a^*	43.3 ± 0.8 *^b^*	nd	nd
Quercetin	59.7 ± 0.8 *^b^*	24.4 ± 0.5 *^c^*	387 ± 3 *^a^*	19.0 ± 0.4 *^c^*
Vanillin	14.8 ± 1.0 *^c^*	18.1 ± 0.5 *^c^*	201 ± 5 *^a^*	56.7 ± 4.2 *^b^*
Syringic acid	9.31 ± 0.41 *^d^*	15.2 ± 0.1 *^c^*	76.6 ± 1.0 *^a^*	44.6 ± 0.9 *^b^*
Gallic acid	3.65 ± 0.08 *^d^*	9.41 ± 0.13 *^c^*	18.9 ± 0.1 *^a^*	10.8 ± 0.6 *^b^*
Ferulic acid	19.35 ± 1.05 *^c^*	7.99 ± 0.15 *^d^*	29.4 ± 0.3 *^b^*	65.1 ± 0.5 *^a^*
Luteolin	133 ± 1 *^b^*	4.55 ± 0.03 *^c^*	176 ± 3 *^a^*	4.90 ± 0.09 *^c^*
*p*-Coumaric acid	12.8 ± 0.4 *^c^*	4.60 ± 0.27 *^d^*	90.4 ± 1.3 *^a^*	22.9 ± 0.5 *^b^*
Eriodictyol	14.24 ± 0.45 *^b^*	1.39 ± 0.03 *^c^*	22.1 ± 2.2 *^a^*	2.86 ± 0.08 *^c^*

Values indicated by the same superscripts (*a*–*d*) within the same row are not significantly different according to Tukey’s HSD test at the 5% significance level. nd: Not detected.

**Table 2:** Antioxidant activities of *Helichrysum noeanum* extracts.

Assays	Flowers	Leaves	Stems	Roots	Trolox	EDTA
Phosphomolybdenum (EC_50_: mg/mL)	0.85 ± 0.01 ^c^	0.70 ± 0.01 ^b^	0.74 ± 0.02 ^b^	0.84 ± 0.02 ^c^	0.45 ± 0.04 ^a^	-
CUPRAC reducing power (EC_50_: mg/mL)	0.82 ± 0.03 ^d^	0.55 ± 0.01 ^b^	0.68 ± 0.01 ^c^	0.82 ± 0.01 ^d^	0.17 ± 0.01 ^a^	-
FRAP reducing power (EC_50_: mg/mL)	0.45 ± 0.01 ^d^	0.27 ± 0.01 ^b^	0.35 ± 0.01 ^c^	0.48 ± 0.01 ^e^	0.05 ± 0.002 ^a^	-
DPPH radical (IC_50_: mg/mL)	2.66 ± 0.06 ^c^	2.10 ± 0.23 ^b^	2.45 ± 0.07 ^bc^	2.76 ± 0.13 ^c^	0.26 ± 0.01 ^a^	-
ABTS radical cation (IC_50_: mg/mL)	1.31 ± 0.01 ^c^	0.96 ± 0.01 ^b^	1.27 ± 0.01 ^c^	1.51 ± 0.05 ^d^	0.18 ± 0.01 ^a^	-
Ferrous ion chelating (IC_50_: mg/mL)	4.10 ± 0.37 ^c^	2.02 ± 0.07 ^b^	3.15 ± 0.40 ^c^	6.98 ± 0.26 ^d^	-	0.019 ± 0.001 ^a^

EDTAE means ethylenediaminetetraacetic acid (disodium salt). Values indicated by the same superscripts (a–e) within the same row are not significantly different according to Tukey’s HSD test at the 5% significance level.

**Table 3:** Enzyme inhibition activity of *Helichrysum noeanum* extracts.

Samples	AChE Inhibition (IC_50_: mg/mL)	BChE Inhibition (IC_50_: mg/mL)	Tyrosinase Inhibition (IC_50_: mg/mL)	α-Amylase Inhibition (IC_50_: mg/mL)	α-Glucosidase Inhibition (IC_50_: mg/mL)
Flowers	5.58 ± 0.02 *^d^*	14.31 ± 0.39 *^c^*	1.20 ± 0.01 *^c^*	3.69 ± 0.04 *^d^*	1.01 ± 0.03 *^a^*
Leaves	1.45 ± 0.09 *^c^*	1.25 ± 0.02 *^b^*	1.14 ± 0.01 *^b^*	2.92 ± 0.04 *^c^*	1.04 ± 0.01 *^a^*
Stems	1.51 ± 0.09 *^c^*	1.24 ± 0.01 *^b^*	1.14 ± 0.01 *^b^*	2.39 ± 0.02 *^b^*	1.11 ± 0.02 *^b^*
Roots	1.00 ± 0.01 *^b^*	1.16 ± 0.01 *^b^*	1.13 ± 0.01 *^b^*	2.81 ± 0.01 *^c^*	1.08 ± 0.01 *^ab^*
Galanthamine	0.0032 ± 0.0001 *^a^*	0.0032 ± 0.0001 *^a^*	-	-	-
Kojic acid	-	-	0.083 ± 0.001 *^a^*	-	-
Acarbose	-	-	-	0.94 ± 0.03 *^a^*	1.13 ± 0.01 *^b^*

Values indicated by the same superscripts (*a*–*d*) within the same column are not significantly different according to Tukey’s HSD test at the 5% significance level.

**Table 4:** Pearson correlation coefficients among total phenolic and flavonoid contents, phenolic compounds, antioxi-dant assays, and enzyme inhibitory activities across the investigated organs of *H. noeanum*.

	TAP	DPPH	ABTS	CUPRAC	FRAP	FICA	AChEIA	BChEIA	TIA	AAIA	AGIA
DPPH radical	0.798										
ABTS radical cation	0.832	0.873									
CUPRAC reducing power	0.932	0.924	0.937								
FRAP reducing power	0.940	0.935	0.947	0.990							
Ferrous ion chelating	0.870	0.880	0.967	0.957	0.963						
RACI	0.908	0.910	0.957	0.961	0.983	0.986					
AChE inhibition	0.212	0.069	−0.127	0.145	0.056	−0.126					
BChE inhibition	0.544	0.298	0.136	0.427	0.357	0.181	0.923				
Tyrosinase inhibition	0.458	0.176	0.034	0.317	0.246	0.069	0.945	0.990			
α-Amylase inhibition	0.516	0.184	−0.023	0.292	0.271	0.126	0.644	0.822	0.810		
α-Glucosidase inhibition	−0.266	0.080	0.286	−0.029	−0.007	0.158	−0.634	−0.723	−0.734	−0.911	
Total flavonoid	0.033	0.063	0.179	0.001	0.103	0.254	−0.899	−0.704	−0.738	−0.266	0.352
Total phenolic	0.925	0.870	0.933	0.955	0.970	0.985	−0.075	0.262	0.155	0.266	0.032
Chlorogenic acid	0.664	0.705	0.824	0.716	0.780	0.866	−0.565	−0.244	−0.347	−0.078	0.303
Hyperoside	0.732	0.548	0.552	0.610	0.674	0.694	−0.289	0.089	0.006	0.457	−0.249
Caffeic acid	−0.547	−0.577	−0.700	−0.576	−0.653	−0.761	0.682	0.369	0.461	0.110	−0.308
Luteolin 7-glucoside	−0.405	−0.224	−0.065	−0.331	−0.249	−0.065	−0.978	−0.971	−0.974	−0.684	0.627
Protocatechuic acid	0.472	0.121	−0.027	0.213	0.239	0.162	0.232	0.487	0.468	0.891	−0.799
3-Hydroxybenzoic acid	0.236	0.084	0.094	0.087	0.176	0.256	−0.585	−0.312	−0.350	0.242	−0.160
4-Hydroxybenzoic acid	0.239	0.098	0.132	0.099	0.195	0.277	−0.640	−0.365	−0.406	0.175	−0.102
Apigenin	−0.569	−0.359	−0.208	−0.482	−0.409	−0.233	−0.923	−0.993	−0.977	−0.759	0.653
Apigenin 7-glucoside	−0.653	−0.434	−0.288	−0.561	−0.496	−0.326	−0.875	−0.988	−0.960	−0.789	0.653
Quercetin	0.302	−0.006	−0.126	0.049	0.099	0.074	−0.039	0.205	0.192	0.722	−0.662
Vanillin	0.264	−0.085	−0.251	−0.010	0.013	−0.059	0.237	0.425	0.431	0.859	−0.824

Data show the Pearson Correlation Coefficients between the parameters. TAP: total antioxidant activity by the phosphomolybdenum method. AAIA, AGAI, AChEIA, BChEIA and TIA: α-amylase, α-glucosidase, acetylcholinesterase, butyrylcholinesterase and tyrosinase inhibition activities, respectively. ABTS and DPPH: ABTS and DPPH radical-scavenging activities, respectively. CUPRAC and FRAP: CUPRAC and FRAP reducing power potential, respectively. FICA: Ferrous ion chelating activity. RACI: Relative antioxidant capacity index. Correlations should be regarded as exploratory observations due to the limited sample size (*n* = 4).

## Data Availability

Data will be made available on request.

## Supplementary Materials

The following supporting information can be downloaded at: https://www.mdpi.com/article/10.3390/molecules31132390/s1, Table S1: ESI–MS/MS Parameters and analytical characteristics for the Analysis of Target Analytes by MRM Negative and Positive Ionization Mode; Table S2: Calibration curves and sensitivity properties of the method.

## References

[1] Lin D., Xiao M., Zhao J., Li Z., Xing B., Li X., Kong M., Li L., Zhang Q., Liu Y. (2016). An overview of plant phenolic compounds and their importance in human nutrition and management of type 2 diabetes. Molecules.

[2] Yordi E.G., Pérez E.M., Matos M.J., Villares E.U. (2012). Antioxidant and pro-oxidant effects of polyphenolic compounds and structure-activity relationship evidence. Nutrition, Well-Being and Health.

[3] Bouzekri O., El Gamouz S., El Idrissi M., Amechrouq A., Choukrad M.B. (2023). Organ-Dependent Variability in Phytochemical Content and Antioxidant Activities of Extracts from Various Parts of *Asteriscus graveolens* and *Brocchia cinerea* (Pearson Correlation). Proc. Natl. Acad. Sci. India Sect. B Biol. Sci..

[4] Lourens A.C.U., Viljoen A.M., Van Heerden F.R. (2008). South African *Helichrysum* species: A review of the traditional uses, biological activity and phytochemistry. J. Ethnopharmacol..

[5] Akinyede K.A., Cupido C.N., Hughes G.D., Oguntibeju O.O., Ekpo O.E. (2021). Medicinal properties and In vitro biological activities of selected *Helichrysum* species from South Africa: A review. Plants.

[6] Akaberi M., Sahebkar A., Azizi N., Emami S.A. (2019). Everlasting flowers: Phytochemistry and pharmacology of the genus *Helichrysum*. Ind. Crops Prod..

[7] Elkiran O., Bagci E., Evren H. (2013). Composition of the essential oil of endemic *Helichrysum noeanum* Boiss. (Asteraceae) growing wild in Turkey. Asian J. Chem..

[8] Kürkçüoğlu M., Ağalar H., Aksoy A., Baser K. (2019). Composition of the essential oils of two endemic *Helichrysum* species in Turkey. Rec. Nat. Prod..

[9] Ak G., Zengin G., Sinan K.I., Mahomoodally M.F., Picot-Allain M.C.N., Cakır O., Bensari S., Yılmaz M.A., Gallo M., Montesano D. (2020). A comparative bio-evaluation and chemical profiles of *Calendula officinalis* L. extracts prepared via different extraction techniques. Appl. Sci..

[10] Kramberger K., Jenko Pražnikar Z., Baruca Arbeiter A., Petelin A., Bandelj D., Kenig S. (2021). A comparative study of the antioxidative effects of *Helichrysum italicum* and *Helichrysum arenarium* infusions. Antioxidants.

[11] Apak R., Güçlü K., Özyürek M., Karademir S.E. (2004). Novel total antioxidant capacity index for dietary polyphenols and vitamins C and E, using their cupric ion reducing capability in the presence of neocuproine: CUPRAC method. J. Agric. Food Chem..

[12] Benzie I.F., Strain J.J. (1996). The ferric reducing ability of plasma (FRAP) as a measure of “antioxidant power”: The FRAP assay. Anal. Biochem..

[13] Kedare S.B., Singh R. (2011). Genesis and development of DPPH method of antioxidant assay. J. Food Sci. Technol..

[14] Re R., Pellegrini N., Proteggente A., Pannala A., Yang M., Rice-Evans C. (1999). Antioxidant activity applying an improved ABTS radical cation decolorization assay. Free Radic. Biol. Med..

[15] Prieto P., Pineda M., Aguilar M. (1999). Spectrophotometric quantitation of antioxidant capacity through the formation of a phosphomolybdenum complex: Specific application to the determination of vitamin E. Anal. Biochem..

[16] Gülçin İ., Huyut Z., Elmastaş M., Aboul-Enein H.Y. (2010). Radical scavenging and antioxidant activity of tannic acid. Arab. J. Chem..

[17] Sun T., Tanumihardjo S. (2007). An integrated approach to evaluate food antioxidant capacity. J. Food Sci..

[18] Anand P., Singh B. (2013). A review on cholinesterase inhibitors for Alzheimer’s disease. Arch. Pharmacal Res..

[19] Tundis R., Loizzo M.R., Menichini F. (2010). Natural products as α-amylase and α-glucosidase inhibitors and their hypoglycaemic potential in the treatment of diabetes: An update. Mini Rev. Med. Chem..

[20] Zolghadri S., Bahrami A., Hassan Khan M.T., Munoz-Munoz J., Garcia-Molina F., Garcia-Canovas F., Saboury A.A. (2019). A comprehensive review on tyrosinase inhibitors. J. Enzym. Inhib. Med. Chem..

[21] Azmir J., Zaidul I.S.M., Rahman M.M., Sharif K.M., Mohamed A., Sahena F., Jahurul M.H.A., Ghafoor K., Norulaini N.A.N., Omar A.K.M. (2013). Techniques for extraction of bioactive compounds from plant materials: A review. J. Food Eng..

[22] Niggeweg R., Michael A.J., Martin C. (2004). Engineering plants with increased levels of the antioxidant chlorogenic acid. Nat. Biotechnol..

[23] Stelzner J., Roemhild R., Garibay-Hernández A., Harbaum-Piayda B., Mock H.-P., Bilger W. (2019). Hydroxycinnamic acids in sunflower leaves serve as UV-A screening pigments. Photochem. Photobiol. Sci..

[24] Bilger W., Johnsen T., Schreiber U. (2001). UV-excited chlorophyll fluorescence as a tool for the assessment of UV-protection by the epidermis of plants. J. Exp. Bot..

[25] Sharma A., Shahzad B., Rehman A., Bhardwaj R., Landi M., Zheng B. (2019). Response of phenylpropanoid pathway and the role of polyphenols in plants under abiotic stress. Molecules.

[26] Les F., Venditti A., Cásedas G., Frezza C., Guiso M., Sciubba F., Serafini M., Bianco A., Valero M.S., López V. (2017). Everlasting flower (*Helichrysum stoechas* Moench) as a potential source of bioactive molecules with antiproliferative, antioxidant, antidiabetic and neuroprotective properties. Ind. Crops Prod..

[27] Carini M., Aldini G., Furlanetto S., Stefani R., Facino R.M. (2001). LC coupled to ion-trap MS for the rapid screening and detection of polyphenol antioxidants from *Helichrysum stoechas*. J. Pharm. Biomed. Anal..

[28] Jaradat N., Qneibi M., Hawash M., Sawalha A., Qtaishat S., Hussein F., Issa L. (2021). Chemical composition, antioxidant, antiobesity, and antidiabetic effects of *Helichrysum sanguineum* (L.) Kostel. from Palestine. Arab. J. Sci. Eng..

[29] Kherbache A., Senator A., Laouicha S., Al-Zoubi R.M., Bouriche H. (2020). Phytochemical analysis, antioxidant and anti-inflammatory activities of *Helichrysum stoechas* (L.) Moench extracts. Biocatal. Agric. Biotechnol..

[30] Bubna G.A., Lima R.B., Zanardo D.Y.L., Dos Santos W.D., Ferrarese M.d.L.L., Ferrarese-Filho O. (2011). Exogenous caffeic acid inhibits the growth and enhances the lignification of the roots of soybean (*Glycine max*). J. Plant Physiol..

[31] Li S., Pi J., Zhu H., Yang L., Zhang X., Ding W. (2021). Caffeic acid in tobacco root exudate defends tobacco plants from infection by *Ralstonia solanacearum*. Front. Plant Sci..

[32] Xie H., Zhou X. (2022). Effects of ferulic and p-hydroxybenzoic acids on Fusarium community structure and abundance in cucumber seedling rhizosphere. Can. J. Soil Sci..

[33] Albayrak S., Aksoy A., Sagdic O., Hamzaoglu E. (2010). Compositions, antioxidant and antimicrobial activities of *Helichrysum* (Asteraceae) species collected from Turkey. Food Chem..

[34] Tepe B., Sokmen M., Akpulat H.A., Sokmen A. (2005). In vitro antioxidant activities of the methanol extracts of four *Helichrysum* species from Turkey. Food Chem..

[35] Acet T., Ozcan K., Zengin G. (2020). An assessment of phenolic profiles, fatty acid compositions, and biological activities of two *Helichrysum* species: *H. plicatum* and *H. chionophilum*. J. Food Biochem..

[36] Güven L., Gülçin İ. (2024). Determination of metabolic profiling by LC-MS/MS, evaluation of antioxidant activities, and enzyme inhibition effects of *Helichrysum plicatum* subsp. *pseudopliacatum*. KSU J. Agric. Nat..

[37] Orhan I., Kartal M., Tosun F., Sener B. (2007). Screening of various phenolic acids and flavonoid derivatives for their anticholinesterase potential. Z. Naturforsch. C.

[38] Khan H., Marya, Amin S., Kamal M.A., Patel S. (2018). Flavonoids as acetylcholinesterase inhibitors: Current therapeutic standing and future prospects. Biomed. Pharmacother..

[39] Yıldız G., Şeker Karatoprak G., İlgün S., Yilmaz M.A., Köse Y.B., Zengin G. (2024). The relationship between the chemical components and skin-related enzyme inhibition, antioxidant activities and toxicity profile of *Helichrysum armenium* subsp. *armenium*. Plant Biosyst..

[40] Popoola O.K., Marnewick J.L., Rautenbach F., Ameer F., Iwuoha E.I., Hussein A.A. (2015). Inhibition of oxidative stress and skin aging-related enzymes by prenylated chalcones and other flavonoids from *Helichrysum teretifolium*. Molecules.

[41] Sarikurkcu C., Locatelli M., Mocan A., Zengin G., Kirkan B. (2020). Phenolic Profile and Bioactivities of *Sideritis perfoliata* L.: The Plant, Its Most Active Extract, and Its Broad Biological Properties. Front. Pharmacol..

[42] Selvi B. (2025). Comparative study of *Pentanema verbascifolium* extracts: Phytochemical composition, antioxidant potential, and enzyme inhibition across plant parts. Food Biosci..

[43] Karakus Z. (2026). Phytochemical Characterisation and Bioactivity of *Picnomon acarna* Extracts: LC–MS/MS Profiling, Antioxidant Capacity and Enzyme Inhibition. Molecules.

[44] Sarikurkcu C., Ozer M.S., Calli N., Popovic-Djordjevic J. (2018). Essential oil composition and antioxidant activity of endemic *Marrubium parviflorum* subsp. *oligodon*. Ind. Crops Prod..

[45] Cittan M., Çelik A. (2018). Development and validation of an analytical methodology based on Liquid Chromatography–Electrospray Tandem Mass Spectrometry for the simultaneous determination of phenolic compounds in olive leaf extract. J. Chromatogr. Sci..

[46] Kose E., Aktas O., Deniz I., Sarikürkçü C. (2010). Chemical composition, antimicrobial and antioxidant activity of essential oil of endemic *Ferula lycia* Boiss. J. Med. Plants Res..

[47] Tlili N., Sarikurkcu C. (2020). Bioactive compounds profile, enzyme inhibitory and antioxidant activities of water extracts from five selected medicinal plants. Ind. Crops Prod..

[48] Saravanakumar K., Sarikurkcu C., Sarikurkcu R.T., Wang M.H. (2019). A comparative study on the phenolic composition, antioxidant and enzyme inhibition activities of two endemic *Onosma* species. Ind. Crops Prod..

[49] Gursoy N., Sarikurkcu C., Tepe B., Solak M.H. (2010). Evaluation of Antioxidant Activities of 3 Edible Mushrooms: *Ramaria flava* (Schaef.: Fr.) Quel., *Rhizopogon roseolus* (Corda) TM Fries., and *Russula delica* Fr. Food Sci. Biotechnol..

[50] Apak R., Güçlü K., Özyürek M., Esin Karademir S., Erçaǧ E. (2006). The cupric ion reducing antioxidant capacity and polyphenolic content of some herbal teas. Int. J. Food Sci. Nutr..

[51] Arumugam R., Kirkan B., Sarikurkcu C. (2019). Phenolic profile, antioxidant and enzyme inhibitory potential of methanolic extracts from different parts of *Astragalus ponticus* Pall. S. Afr. J. Bot..

